# Differences in home food availability of high- and low-fat foods after a behavioral weight control program are regional not racial

**DOI:** 10.1186/1479-5868-7-69

**Published:** 2010-09-24

**Authors:** Rebecca A Krukowski, Jean Harvey-Berino, Delia Smith West

**Affiliations:** 1 Fay W. Boozman College of Public Health, University of Arkansas for Medical Sciences, Little Rock, AR, USA; 2 Department of Nutrition and Food Sciences, University of Vermont, Burlington, VT, USA

## Abstract

**Background:**

Few studies, if any, have examined the impact of a weight control program on the home food environment in a diverse sample of adults. Understanding and changing the availability of certain foods in the home and food storage practices may be important for creating healthier home food environments and supporting effective weight management.

**Methods:**

Overweight adults (n = 90; 27% African American) enrolled in a 6-month behavioral weight loss program in Vermont and Arkansas. Participants were weighed and completed measures of household food availability and food storage practices at baseline and post-treatment. We examined baseline differences and changes in high-fat food availability, low-fat food availability and the storage of foods in easily visible locations, overall and by race (African American or white participants) and region (Arkansas or Vermont).

**Results:**

At post-treatment, the sample as a whole reported storing significantly fewer foods in visible locations around the house (-0.5 ± 2.3 foods), with no significant group differences. Both Arkansas African Americans (-1.8 ± 2.4 foods) and Arkansas white participants (-1.8 ± 2.6 foods) reported significantly greater reductions in the mean number of high-fat food items available in their homes post-treatment compared to Vermont white participants (-0.5 ± 1.3 foods), likely reflecting fewer high-fat foods reported in Vermont households at baseline. Arkansas African Americans lost significantly less weight (-3.6 ± 4.1 kg) than Vermont white participants (-8.3 ± 6.8 kg), while Arkansas white participants did not differ significantly from either group in weight loss (-6.2 ± 6.0 kg). However, home food environment changes were not associated with weight changes in this study.

**Conclusions:**

Understanding the home food environment and how best to measure it may be useful for both obesity treatment and understanding patterns of obesity prevalence and health disparity.

## Background

In recent years, efforts to understand the obesity crisis have focused attention on measuring and understanding the role of the "built environment" (i.e., the physical surroundings that may impact dietary intake or energy expenditure, including homes [1]). Many experts now agree that the built environment must be considered in any effort to understand or reduce obesity [2]. Research has begun emerging on possible associations between the home food environment and food intake [3-8]. Studies have found a relationship between number of high-fat foods in the home and fat intake [5] and fruit and vegetable availability in the home and intake of these foods among adolescents [4,6,7]. Understanding home food availability is crucial in fully grasping overall patterns of dietary intake, as Nielsen and colleagues [9] estimated that 65% of all calories were consumed in the home.

Few studies, however, have examined the impact of a behavioral weight control program on the home food environment of adults [10,11], and no studies have examined whether home environment characteristics change over the course of a weight control program in distinct ways for different race groups or in different regions. Ample evidence exists to document differential response to weight loss programs by race [12-15], with African Americans tending to lose less weight than white participants. There is also some suggestion that individuals from different regions of the United States may respond differently to similar weight loss programs, due to higher initial body weight [16,17] or greater initial dietary fat intake [18,19].

Changes in availability of higher fat foods in the home and food storage practices may be important for supporting weight management efforts. Targeting fat intake in addition to overall caloric intake in a behavioral weight control program is strongly supported by studies [20,21] that demonstrate when fat and calories are both controlled, weight loss is improved. Furthermore, some initial evidence suggests an association between successful weight loss and significant changes in the home food environment [10]. Such changes in the food environment within the home may even facilitate weight loss among individuals in the household who are not directly engaged in a weight control intervention program [10]. Thus, parameters associated with healthy changes in the home food environment may be a promising avenue for direct intervention, supporting weight management efforts and promoting healthier dietary intake in general. There is some evidence that the broader food environment differs for African Americans compared with Caucasians[22-24], and it is likely that the broader food environment differs between regions due to greater rural areas in the southern portion of the United States [25]. However, even though the home environment is likely influenced by neighborhood disparities in food availability [3], there have been no explorations to date of regional or racial influences on more immediate home food environments.

The current study prospectively examined changes in food availability and storage of foods in the homes of individuals enrolled in a behavioral weight loss program over six months. Specifically, we examined changes in high-fat food availability, low-fat food availability and the storage of foods in visible locations, overall and by race (African American or white participants) and region (Arkansas or Vermont). We also explored whether changes in the home food environment were related to weight loss achieved in the behavioral weight control program. Examining the home food environment following a weight control program may help to understand the process of lifestyle changes that may be associated with successful weight loss, as well as possible cultural factors (i.e., race and/or region) that may influence these changes.

## Methods

### Participants

Participants were recruited in 2007-2008 for a behavioral weight loss research program through community-based efforts such as presentations, flyers, and social networking and targeted mailings over the Internet. Interested participants completed an online screening and two in-person assessments prior to randomization. Eligibility criteria included overweight (body mass index [BMI] between 25 and 50 kg/m^2^), generally healthy and able to walk for exercise. Individuals were considered ineligible if they were younger than 18 years, had medical conditions that contraindicated participation in a behavioral weight loss program containing an exercise component, were pregnant or lactating, or were enrolled in another weight reduction program. Participants lived within 45 minutes by car of Little Rock, Arkansas or Burlington, Vermont. These regions encompass a mix of urban, suburban, and rural areas. Individuals enrolled in the final wave of recruitment (N = 90) for a larger randomized controlled trial were eligible for this ancillary study. All participants received the same 6-month behavioral weight control program. The study was approved by the Committee on Human Research in the Behavioral Sciences at the University of Vermont and the Institutional Review Board at the University of Arkansas for the Medical Sciences.

### Measures

Participants completed assessments including the following measures at baseline and after 6 months of a behavioral weight control program:

#### Demographic Characteristics

Participants reported their gender, age, education and race.

#### Weight and Height

Participants were weighed in street clothes without shoes on a calibrated digital scale. Height was assessed using a wall-mounted stadiometer. BMI was calculated as weight (kg)/height (m)^2^.

#### Household Food Environment

The *Household Food Inventory *[8,10,11] was used to quantify household food availability based on 26 common food categories that included 14 high-fat food categories (> 45% calories from fat) (e.g., cheese, cookies, beef/pork/lamb) and 12 low-fat food categories (< 18% calories from fat) (e.g., broccoli, tomatoes/tomato juice [including pico de gallo], apples/applesauce/pears). Participants were instructed to "check all places where food might be stored and indicate whether each food category was available regardless of the amount on that day." Previous research has found good test-retest reliability (Pearson *r *correlations > 0.70) [8] and inter-rater reliability (i.e., concordance with spouse) [10] for this measure. Furthermore, Raynor and colleagues [8] found a strong association between the availability of food items on this measure and dietary fat intake. The measure has been used previously in an ethnically-diverse population with subjects from both northern and southern regions of the United States and was demonstrated in that study to be correlated with actual dietary intake, as well as sensitive to changes in the home food environment following a behavioral weight control program [10]. To further characterize the home food environment, the *Food Storage Questionnaire *[26] was used to assess food storage practices in the home. Participants were instructed to "look around their homes, without opening any cabinets, drawers, the refrigerator, or the freezer, and indicate whether foods from 11 categories of healthier and less healthy foods (e.g., dried fruits, chocolate, nuts and seeds, pie) were stored in readily visible locations (e.g., on countertops, kitchen table, living room coffee table)." At baseline only, participants were asked to indicate how many days it had been since their last food shopping trip.

### Intervention

A comprehensive behavioral weight loss program targeting dietary restriction and physical activity promotion, as well as behavioral strategies to achieve the diet and activity change recommendations, was offered to all participants over 24 weekly one-hour group sessions. All sessions were delivered by a trained facilitator with expertise in conducting behavioral weight control interventions and were based on empirically established programs [27-29]. Participants were instructed to strive for a weight loss of 7% or more of initial weight. They were given a daily calorie and fat intake goal to help them achieve this weight loss. Weekly aerobic exercise goals progressed from 50 to 200 minutes over nine weeks and remained at 200 minutes weekly thereafter. Behavioral strategies introduced to facilitate and support recommended habit changes included goal setting, problem solving, reducing food cues, and self monitoring. However, home food environment changes were only a minor aspect of the program and included recommendations to remove visible food cues from the home by storing foods out of sight, to increase the availability of low calorie, low-fat foods readily accessible in the house and to modify grocery shopping behaviors. Participants were asked to self monitor daily dietary intake and physical activity, as well as to weigh themselves daily. Self monitoring was reviewed by the facilitator and written feedback was provided on a weekly basis. A high rate of adherence to the study goals of attendance and self-monitoring were achieved: participants submitted 73% of self-monitoring journals and attended an average of 69% of their group meetings [30].

### Analysis

Repeated measures t-tests were used to examine overall changes over the treatment period in home food environment variables. Race and region-based group differences (i.e., three groups-- Arkansas African American participants, Arkansas white participants, Vermont white participants) in baseline weight and weight change over treatment were examined using analysis of variance (ANOVA). Post-hoc comparisons were conducted using Tukey's HSD test. In addition, race and region-based group differences were examined using between-groups analysis of covariance (ANCOVA), controlling for baseline weight. Between-groups ANCOVA was also used to assess the group differences in the impact of a behavioral weight control program on the home food environment, controlling for weight change over the treatment period. Relationships between home food environment variables and baseline weight and weight change were examined using Pearson correlations. In addition, Pearson correlation were used to examine the relationship between baseline home food environment and the number of days since the participant had last gone food shopping. To account for participants who were missing some or all data at the post-treatment data collection visit (n = 16), baseline observation was carried forward (i.e., baseline value was inserted for the post-treatment value). This method is preferred for interpolating weight change data [31]. Data were analyzed using SPSS 16.0, using a significance level of 0.05.

## Results

Participants (n = 90) were predominantly obese (BMI range: 27.7-49.1), with a mean age of 44.6 years (range: 18- 65 years) and were 95% female, with 73% self identifying as white and 27% as African American (Table 1). All of the African American participants were enrolled from Arkansas. The racial proportions of the sample recruited in Arkansas were 54% African American and 46% white. The majority of the participants in the study (65%) had at least a college degree. Significantly more Arkansas African Americans (69%) and Vermont white participants (69%) had at least a college degree as compared to the Arkansas white participants (50%). There was a statistically significant difference in baseline weight for the three race- and region-based groups. Post-hoc comparisons indicated that Arkansas African Americans were significantly heavier than Vermont white participants. Arkansas white participants did not differ significantly from either the Arkansas African Americans or the Vermont white participants in baseline weight. There were no significant differences in age and proportion female between the race and region-based groups.

**Table 1:** Baseline Demographic and Unadjusted Home Food Environment Characteristics*

	Overall N = 90	Vermont White participants N = 44	Arkansas White participants N = 21	Arkansas African American N = 25	p-value
Age (years)	44.6±10.2	46.2±10.2	42.7±11.4	41.7±2.5	*p *> 0.05
Female (%)	95%	93%	96%	100%	*p *> 0.05
					
College Education or Higher (%)	65%	69%	50%	69%	***p*< 0.05**
Weight (kg)	98.3±19.0	94.0±20.3	98.0±13.5	106.0±18.8	***p*< 0.05**
Food Storage (FSQ^a ^food categories)	3.6±2.5	3.2±2.2	3.7±2.1	4.1±3.1	*p *> 0.05
Low-Fat Foods (HFI^b ^food categories)	7.2±2.1	7.6±1.9	6.4±2.1	7.0±2.1	*p *= 0.075
					
High-Fat Foods (HFI^b ^food categories)	8.5±2.5	7.6±2.0	9.4±3.0	9.2±2.5	***p*< 0.01**

* (Mean ± SD unless otherwise noted); ^a^Food Storage Questionnaire; ^b^Household Food Inventory

There were no significant baseline differences between Arkansas African Americans, Arkansas white participants, and Vermont white participants in baseline food storage practices in the home (see Table 1). However, there were significant differences between the three groups in the number of high-fat food availability in the home at baseline. Post-hoc comparisons indicate that the mean number of high-fat foods that the Vermont white participants reported was significantly lower than both the mean number of household high-fat foods reported by both Arkansas white participants and Arkansas African American participants. There was a marginally significant difference between the groups in the number of low-fat foods in the home, such that there was a non-significant trend for Vermont white participants to have more low-fat foods in their homes than both the Arkansas white participants or African American participants, with no difference between Arkansas groups. To account for baseline differences in weight of the three race- and region-based groups, the baseline analyses for the food environment variables (availability and storage) were repeated using an ANCOVA to adjust for baseline weight. Similar results were found, so unadjusted values are presented in Table 1. There was no significant relationship between any of the baseline home food environment variables and the number of days since the last food shopping trip. No significant correlation was observed between baseline self-report of any of the home food environment measures and baseline weight in the sample as a whole or within any of the race-region groups.

At post-treatment, there was a statistically significant difference in weight loss across the three groups. Post-hoc comparisons indicated that Arkansas African Americans lost significantly less weight than Vermont white participants. Arkansas white participants had intermediate weight loss outcomes which did not differ significantly from either the Arkansas African Americans or the Vermont white participants (Table 2).

**Table 2:** 6-month Changes in Weight and the Home Food Environment (Mean ± SD)

	Overall N = 90	Vermont White participants N = 44	Arkansas White participants N = 21	Arkansas African American N = 25	p-value
Weight Change (kg)	-6.5±6.2	-8.3±6.8	-6.2±6.0	-3.6±4.1	***p*< 0.01**
Food Storage Change (FSQ^a ^food categories)	-0.5±2.3	-0.8±2.0	-0.2±2.6	-0.3±2.6	*p *> 0.05
Low-Fat Foods Change (HFI^b ^food categories)	-0.06±1.7	-0.1±1.4	-0.05±1.7	0.04±2.1	*p *> 0.05
High-Fat Foods Change (HFI^b ^food categories)	-1.1±2.1	-0.5±1.3	-1.8±2.6	-1.8±2.4	***p *< 0.01**

^a^Food Storage Questionnaire; ^b^Household Food Inventory

At post-treatment, the sample as a whole reported storing significantly fewer foods in visible locations around the house (*p *< 0.05). They also reported a significant reduction in the number of high-fat foods in their homes (*p *< 0.001) from baseline to post-treatment; however, they did not report a significant change in low-fat food availability (*p *> 0.05) over this time period. When examining changes in food storage and availability in the race-region groups, there were no significant differences between groups in self-reported changes in food storage practices or low-fat food availability. However, there was a significant difference in the self-reported changes in high-fat food availability in the homes of Arkansas African Americans, Arkansas white participants, and Vermont white participants. Both Arkansas African Americans and Arkansas white participants reported significantly greater reductions in the mean number high-fat food items available in their home post-treatment compared to Vermont white participants (with no significant difference between Arkansas African Americans and Arkansas white participants). To account for group differences in weight change, these analyses regarding changes in the food environment variables were repeated using an ANCOVA to adjust for difference in weight change; similar results were found, so unadjusted values are presented in Table 2. However, there were no significant associations observed between pre- to post-treatment weight loss and change in any of the home food environment measures over this period.

## Discussion

Consistent with treatment recommendations to reduce fat intake and remove food cues, participants completing a 6-month behavioral weight loss program reported reducing the availability of high-fat foods in their homes and storing fewer foods in visible locations. There were no significant changes in the number of low-fat foods they reported available in their homes. Changes in high-fat food availability in the home found in the current study were similar in magnitude to those reported in previous research using the same measure [10]. In the two available studies that similarly examined changes in availability [10,11], a significant reduction in the availability of high-fat foods was also reported among participants in behavioral weight control programs (mean decrease of approximately 0.8 high-fat foods in both studies). In contrast to one of these previous studies [10], home food environment changes were not associated with weight changes in the current study. A significant correlation between changes in food availability and weight loss was reported over the 12-month period they studied [10]. It may be that home food environment changes require a longer period of time to influence weight loss than the 6 month period examined in the current study or the 8-week period examined in Gorin and colleagues in their earlier study [11]. On the other hand, a greater magnitude of change to the home environment, as was demonstrated when home grocery delivery was provided in the earlier Gorin et al. study [11], may be required to influence weight loss. Alternatively, the absence of a correspondence between self-reported changes in home food environment and weight change may reflect social desirability (i.e., a reporting bias). All participants in the study were in an intervention that counseled reductions in high-fat foods; therefore, some biases toward reporting decreased high-fat foods in the house might be expected. However, the lack of evidence of changes in the presence of low-fat foods in the home offers some evidence that the impact of a social desirability bias was modest since participants were also counseled to increase their consumption of low-fat, low-calorie foods and a similar effect would therefore have been expected with this variable.

The assessment of home food environment clearly warrants further examination in the context of supporting behavioral weight control efforts. Such research efforts would likely benefit from refined methods to measure the home food environment since current measures are fairly limited and fail to reflect the complicated, multi-factorial nature of the overall home food environment. This concern has been raised about measures of the built environment in general [1] and likely applies to the home food environment in particular. There are likely other crucial factors in the home food environment that may be associated with weight loss which are not captured in the currently available assessment methods [32]. Indeed, it may be important to take a step back and actively work on developing and evaluating standardized measures tailored to evaluating the home food environment prior to undertaking further research in the arena of weight management and home food environment. At this point, the lack of standardized home food availability measures and methodology limits the ability to compare research on home food environments [4-8,10,11,32].

Continued research exploring race/ethnicity and regional elements of the home food environment associated with weight management offers potential for significantly enhancing the understanding of obesity development and treatment. Differences in environmental factors may be contributing to observed differences in the prevalence of obesity [16,17,33] and in obesity treatment outcomes [12-15]. It may be important to take a broader approach to the study of the home environment, as food intake and household food availability are influenced by other spheres including the food environment of the community [3].

A greater understanding racial/ethnic and regional differences in food availability in the home would be advantageous given that studies suggest that household availability may impact obesity prevalence and food consumption for multiple household members [10,34]. Furthermore, modifications to the micro-level food environment (i.e., the home) may be more feasible for individuals interested in addressing obesity rather than re-structuring the larger macro-level food environment (i.e., restaurants, supermarkets). The potential for enhancing the success of behavioral weight control approaches by more directly and robustly targeting modifications to the household food environment is intriguing and worthy of further exploration as it offers a promising direction for refining available obesity treatment methods. Thus, a greater focus on the micro-level food environments may be useful for both obesity treatment and understanding patterns of obesity prevalence.

Some researchers have suggested that cultural factors play an important and often ignored role in the development of obesity [35], and the current study would suggest that regional factors might also be considered. The overlap between the cultural factors that characterize the southern region of the United States and those that are common among African American groups has been noted [36,37]. Future considerations of cultural factors associated with obesity might benefit from attention to meaningful regional variations in the cultural meanings and salience of home food environment.

This study has some limitations, including the small sample size, the limited regional representation and the focus on individuals who are engaged in a weight control program. Further, there was racial diversity in only one research center, precluding analyses of a true interaction between race and region. In addition, the lack of information on household composition precluded examining whether the number of individuals in the home made a difference in the home food environment or in the ability to change the home environment as part of a behavioral weight control program. However, the findings are suggestive of potentially important environmental factors that may be salient for understanding the higher dietary fat intake among southerners [18,19] as well as the higher rates of obesity in the south [16,17] and among African Americans [33].

## Conclusions

This study illustrated the largely self-initiated changes in the home food environment of participants enrolled in a 6-month behavioral weight control study. While this study did not demonstrate associations between weight loss and home food environment changes, interesting regional differences in the home food environment did emerge. The role of the home food environment in these regional and racial weight and related health disparities needs further research, as do the possibilities for designing more sensitive measures of the home food environment and methods of modifying this micro-level environment to reduce obesity in these high risk populations.

## Competing interests

The authors declare that they have no competing interests

## Authors' contributions

DSW and JHB obtained the finances for this study and developed and executed the overall study, while RAK oversaw the implementation of the home environment inventories. DSW advised on the conceptions of data collection and analyses. RAK performed the analyses. RAK wrote the drafts of the paper, and JHB and DSW provided critical feedback on drafts. All authors have seen and approved of the version to be published.
